# Revisional endoscopic sleeve gastroplasty for failed laparoscopic sleeve gastrectomy in an immunocompromised patient

**DOI:** 10.1055/a-2689-3271

**Published:** 2025-09-09

**Authors:** Jonathan Rozenberg, Vivek Kesar, Patrick Okolo, Varun Kesar

**Affiliations:** 1 Department of Internal Medicine, Virginia Tech Carilion, Roanoke, United States; 2 Department of Internal Medicine, Division of Gastroenterology, Virginia Tech Carilion, Roanoke, United States

We present a case of a 48-year-old woman with a pertinent past medical history of laparoscopic sleeve gastrectomy (roughly seven and a half years prior) for treatment of class three obesity, rheumatoid arthritis on infliximab, and pyrosis-predominant gastroesophageal reflux disease who presented for endoscopic management of weight recidivism despite surgical bariatric therapy. Of the initial 33.3 kg lost, she had regained 30.8 kg. Staging esophagogastroduodenoscopy (EGD) revealed postsurgical sleeve dilation of the gastric body ( Fig. 1 ). She underwent revisional endoscopic sleeve gastroplasty (r-ESG), which incorporated a “U → I → U → I” suture pattern ( Fig. 2 , Fig. 3 , Fig. 4 ). Seven months post r-ESG, surveillance EGD demonstrated persistent narrowing of the gastric body and some widening at the distal end ( Fig. 5 ) with the patient down 10.8 kg.

**Fig. 1:**
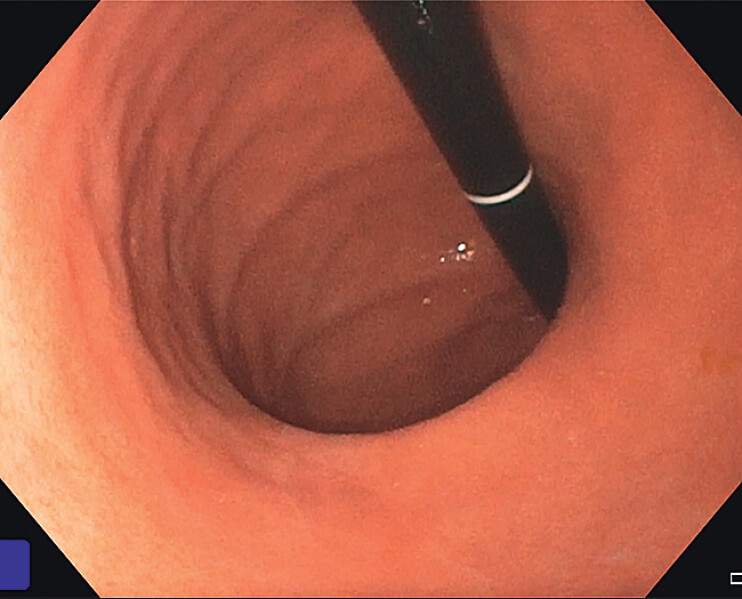
Endoscopic image of a dilated gastric body with known gastric sleeve anatomy in a retroflexed view on esophagogastroduodenoscopy (EGD).

**Fig. 2:**
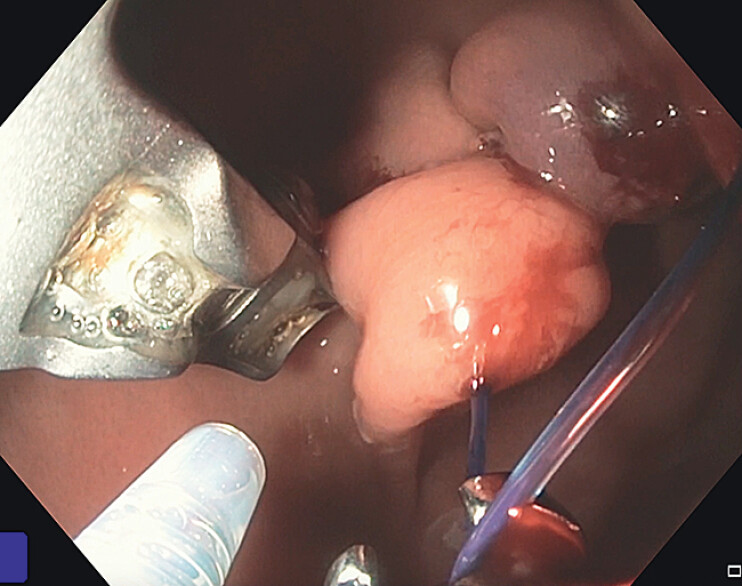
Endoscopic image depicting endoscopic suturing in a “U”-shaped pattern.

**Fig. 3:**
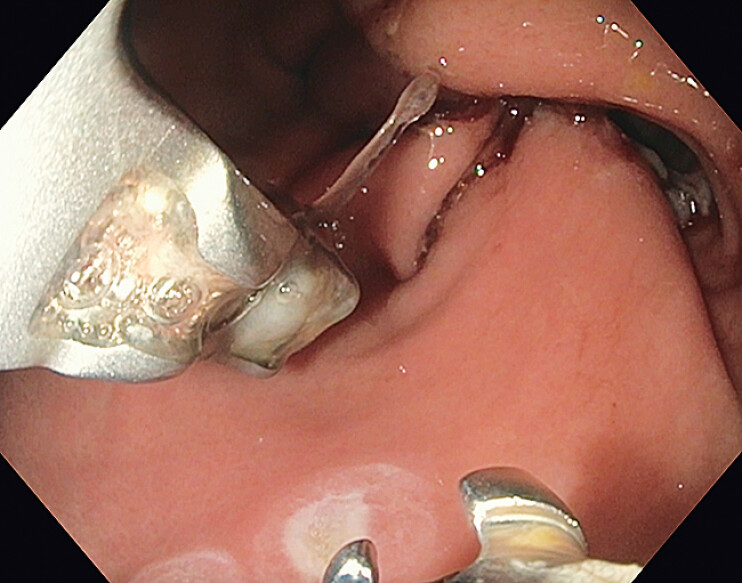
Endoscopic image post cinching of an “U”-shaped suture.

**Fig. 4:**
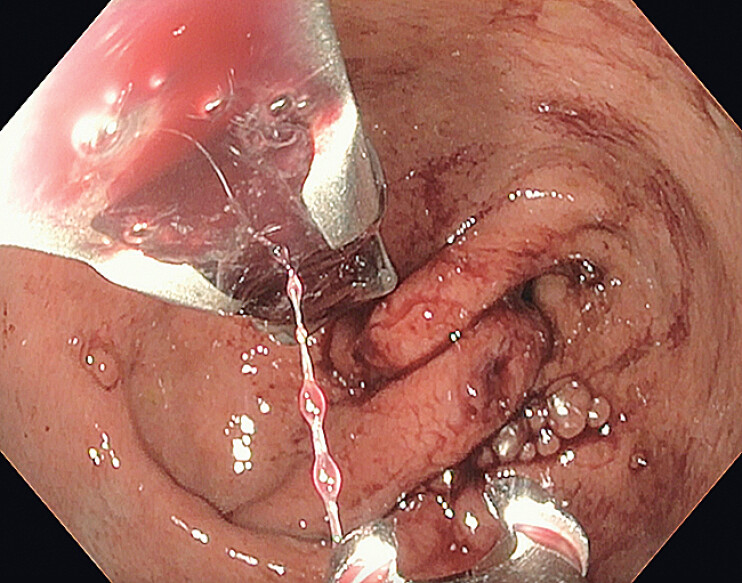
Endoscopic image of the gastric body after placement of an “I”-shaped suture with subsequent gastric body narrowing.

**Fig. 5:**
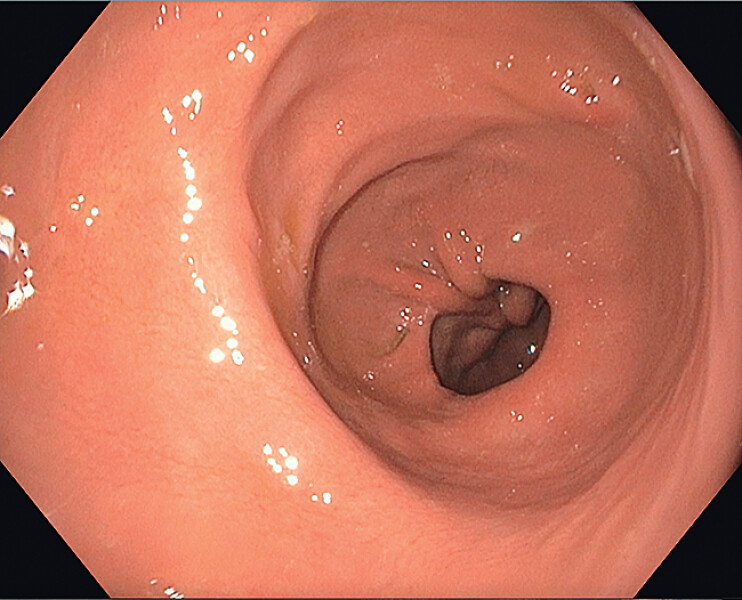
Endoscopic image of the gastric body with persistent narrowing on surveillance EGD at seven months after revisional endoscopic sleeve gastroplasty.

Laparoscopic sleeve gastrectomy (LSG) serves as a common modality for the management of obesity; however, its long-term (≥7 years) efficacy is questionable given the incidence of weight regain/recidivism (<50% excess weight loss) is approximately 27.8% with a range of 14%–37% 1 . While no consensus regarding optimal revisional management of LSG exists, revisional surgical bariatric therapy has shown increased risk – varying from 5% to 20% – for adverse events and higher overall morbidity relative to initial surgical bariatric therapy 2 . Despite limited sample size(s), several studies have indicated that r-ESG provides sustained weight loss at the one- and two-year intervals for post-LSG weight regain 2 3 . As minimal literature exists regarding this topic, little is known about its effectiveness in atypical cases. Thus, this case depicts successful management of refractory obesity due to post-LSG weight recidivism in an immunocompromised patient via r-ESG ( Video 1 ).

Treatment of post-laparoscopic sleeve gastrectomy weight recidivism via revisional endoscopic sleeve gastroplasty in an immunocompromised patient.Video 1

Endoscopy_UCTN_Code_TTT_1AO_2AN

## Notes

Endoscopy E-Videos https://eref.thieme.de/e-videos
*E-Videos* is an open access online section of the journal *Endoscopy* , reporting on interesting cases and new techniques in gastroenterological endoscopy. All papers include a high-quality video and are published with a Creative Commons CC-BY license. Endoscopy E-Videos qualify for HINARI discounts and waivers and eligibility is automatically checked during the submission process. We grant 100% waivers to articles whose corresponding authors are based in Group A countries and 50% waivers to those who are based in Group B countries as classified by Research4Life (see: https://www.research4life.org/access/eligibility/ ). This section has its own submission website at https://mc.manuscriptcentral.com/e-videos .
